# *Tuning in to Toddlers*: Research Protocol and Recruitment for Evaluation of an Emotion Socialization Program for Parents of Toddlers

**DOI:** 10.3389/fpsyg.2019.01054

**Published:** 2019-05-16

**Authors:** Sophie S. Havighurst, Christiane E. Kehoe, Ann E. Harley, Ameika M. Johnson, Nicholas B. Allen, Rae L. Thomas

**Affiliations:** ^1^Mindful: Centre for Training and Research in Developmental Health, Department of Psychiatry, The University of Melbourne, Melbourne, VIC, Australia; ^2^Department of Psychology, Faculty of Social Sciences, University of Oslo, Oslo, Norway; ^3^Department of Psychology, University of Oregon, Eugene, OR, United States; ^4^Centre for Research in Evidence-Based Practice, Bond University, Gold Coast, QLD, Australia

**Keywords:** toddlers, parenting, emotion socialization, emotion coaching, prevention, protocol

## Abstract

**Background:** Parenting a toddler is a challenging experience for many parents with times of emotional dysregulation in both parent and child. Parenting interventions may be useful for parents to improve their ability to regulate emotions and respond to children’s emotions in a way that assists the child to understand and regulate emotions (emotion competence). *Tuning in to Toddlers* (TOTS) is a new parenting program that aims to improve parents’ emotion regulation, emotional responsiveness, and emotion coaching (aspects of emotion socialization) to promote optimal emotional development in toddlers, and prevent social and behavioral difficulties. This paper outlines the rationale, methodology, intervention, and recruitment used in a trial to establish program efficacy.

**Methods/Design:** Parents of toddlers aged 18–36 months old were recruited through child care centers (CC) and maternal child health (MCH) centers in Melbourne, Australia and were allocated to either intervention or a 15-month wait-list control condition in a cluster-randomized controlled design. Inclusion criteria were a child in the age range at baseline attending one of the CC or MCH centers. Exclusion criteria were if the parent/carer had insufficient English to attend the intervention and complete measures. Parents in the intervention condition participated in the 6-session group TOTS program delivered by two facilitators using a structured manual and measures of program fidelity and acceptability. Participants in the wait-list control condition received the intervention after a 15-month waiting period. Participants completed measures at baseline, post-intervention (intervention participants only) and 15-month follow-up. Primary outcome measures included parent emotion socialization (parent-report and observed). Secondary outcomes included parent-reported parent functioning (emotion regulation and mental health), toddler social, emotional and behavioral functioning, and parent and toddler systemic cortisol stress (using hair samples). The study was designed to comply with the CONSORT statement and intervention reporting outlined using TIDieR.

**Results:** Three hundred and six parents were recruited and completed baseline parent questionnaires, with a further 234 completing parent–child observation assessments, 235 parent cortisol, and 198 child cortisol.

**Discussion:** This paper is a methodological description of the TOTS randomized controlled trial evaluation protocol. It outlines some of the challenges in recruiting parents of toddlers to parenting programs.

**Clinical Trial Registration:** www.ClinicalTrials.gov, identifier ACTRN12615000 962538.

## Introduction

The first 3 years of life are a critical and sensitive period for promoting children’s brain development (Sroufe, 1997), as well as their emotional competence (skills in understanding and regulating emotions; Denham, 1998). The parent–child relationship is central in facilitating all aspects of children’s functioning (Mahoney, 2009) and parent emotion socialization is one of the most established factors contributing to children’s emotional competence (Eisenberg et al., 1998). Emotion socialization includes parent’s own expressiveness, and their reactions to and coaching of children’s emotions (Eisenberg et al., 1998). For preschool- and school-aged children, emotion coaching parenting has been linked to better emotion knowledge, social skills and academic results, and fewer physical illnesses and behavior problems (Gottman et al., 1996, 1997; Johnson et al., 2017). Conversely, emotion-dismissing parenting (where parents avoid or are critical of children’s emotions) has been linked to deficits in children’s emotion knowledge and social skills, and more behavior problems (Eisenberg et al., 1998; Johnson et al., 2017). The toddler years are when the foundations for emotional competence are being laid (Denham, 1998), while also being a stressful, challenging time for parents as toddlers often express intense emotions as they strive for independence (Sroufe, 1997). The way parents manage their own emotions and how they respond to their toddler’s emotions is important for reducing stress, promoting optimal development and preventing problems in children (Mahoney, 2009). *Tuning in to Toddlers* (TOTS) is a new program teaching parents these skills but has not yet been evaluated using a randomized controlled trial to determine the impact of the program on parents and toddlers. The following study protocol outlines how TOTS will be evaluated in a randomized controlled trial and also addresses challenges in recruitment of parents of toddlers to a parenting program.

### Background

Central to positive developmental outcomes are parental responsiveness, warmth and sensitivity, combined with an absence of angry, irritable parental affect (Landry et al., 2000). To achieve key developmental milestones in the toddler years, parents need to balance providing boundaries for children’s exploration with being emotionally sensitive and attuned (Belsky and Fearon, 2002). Indeed, the development of an emotionally secure attachment early in life is crucial for healthy social, emotional and behavioral development, with long lasting consequences (Fraley, 2002). Secure attachments are created by countless interactions between parents and very young children within an environment of acceptance, acknowledgment and emotional attunement (van der Voort et al., 2014). Despite this evidence, many prevention or early intervention programs do not start until the preschool or school aged years when problems may have already emerged. There are few evidence-based group programs for parents of toddlers (see Barlow et al., 2016), and those that do exist either focus on changing children’s behavior or work with the parent–child relationship in clinical populations where there are attachment difficulties. Because an emotionally responsive parent–child relationship provides a solid protective base for optimal child development, it is important that early prevention efforts target the parent–child relationship in infancy/toddlerhood when this relationship is still forming. Caring for very young children can also be highly stressful for parents, resulting in greater reactivity in parents and more emotionally dismissive responses with their toddlers (Martorell and Bugental, 2006). Helping parents manage their stress, regulate emotions and parent effectively becomes an important target for prevention.

*Tuning in to Toddlers* (TOTS; Havighurst et al., 2017) is designed to teach parents skills that improve their capacity to manage stress, regulate their emotions, improve parent–child emotional connection, help children understand and regulate their emotions, and ameliorate behavior difficulties early (Sanson et al., 1993). TOTS aims to provide parents with skills in how to emotion coach their toddler; knowledge about toddler development; opportunities to explore automatic beliefs about emotions that may contribute to emotionally dismissive and/or harsh parenting; skills in responding to attachment and exploration needs in their toddler; strategies to help children learn to understand and regulate emotions; and skills in understanding and managing parents own emotions. The TOTS program was adapted from the evidence-based *Tuning in to Kids* parenting program (Havighurst et al., 2010). An initial pilot study of TOTS with 34 parents of 24- to 36-month-old children showed that post-intervention there were reported and observed improvements in parents’ use of emotion coaching and a reduction in emotion dismissing behaviors with medium to large effect sizes; parents also reported fewer toddler disruptive behaviors with small effect sizes (Lauw et al., 2014). Program attendance and satisfaction with the TOTS program was high and the greatest challenge was in recruitment of the target sample. The current study aims to use a randomized controlled design with a larger sample and a control group to more rigorously test whether the TOTS program is efficacious. Efforts to recruit through a number of avenues (childcare and maternal child health centers) were also used to explore whether this increased uptake and engagement.

The proposed study uses parent-report measures and direct observation of parent–toddler interactions to determine any changes in parenting including parent emotion regulation, emotion dismissing, emotion coaching, and harsh/warm parenting. In addition, biological measures of stress are also used to examine whether the intervention leads to reduced parent and toddler stress. Stress can be measured via assessing cortisol, a glucocorticoid hormone (Wells et al., 2014). The hypothalamic–pituitary–adrenal (HPA) axis is activated under stressful conditions releasing cortisol from the adrenal cortex (Staufenbiel et al., 2013). Usually stress has been measured using cortisol concentrations in saliva, blood and urine at a single time point (Wells et al., 2014), however, a newly established technique using hair cortisol concentrations provides a measure of retrospective chronic stress (Russell et al., 2012). While there are some mixed conclusions about the use of hair cortisol as a measure with very young children (Bryson et al., 2019), higher hair cortisol has been found to be associated with chronic stress including chronic pain, unemployment, shift work, serious life events, and psychopathology in adults (Staufenbiel et al., 2013). In order to examine whether TOTS reduced stress in parents and toddlers, the study included hair samples to measure systemic cortisol with the expectation that those in the intervention condition would have lowered parental and toddler stress after the program.

### Aims and Hypotheses

This paper outlines the research protocol and recruitment process being used to evaluate the efficacy of TOTS. This paper also describes the process of recruitment being used to obtain the sample while outlining strategies used to overcome barriers to engaging parents of young children in a parenting intervention. The primary outcome is change on the measures of parent emotion socialization, and the secondary outcomes are change on the measures of parent functioning (emotion regulation and mental health), toddler’s social, behavioral and emotional functioning, and systemic cortisol stress in parents and toddlers. It is hypothesized that based on the TOTS pilot study (Lauw et al., 2014), at 15-month follow-up we will see improved parent emotion socialization (reduced emotion dismissiveness and increased emotion coaching) in intervention participants but not control participants. It is also hypothesized that at 15-month follow-up we will see improved parent functioning (reduced emotion regulation difficulties, improved parent mental health) and reduced child behavior problems in intervention participants but not control participants. The impact of the program has not previously been examined with cortisol stress, however, if parents improve in skills regulating their own emotions and their responses to their toddler’s emotions after program participation, it is hypothesized that at 15-month follow-up there will be lower cortisol stress for parents and toddlers in the intervention condition (but not control).

## Materials and Methods

### Study Design

The study is a cluster randomized controlled design, with three data collection points. Pairs of child care (CC) centers and maternal child health centers (MCH) were matched based on location, socio-economic criteria (i.e., median house price in that area) and parent uptake rate after recruitment. These pairs were then randomized into one of two conditions: intervention or a 15-month wait-list control. A group-randomized design was used, as it is preferable when delivering an intervention to groups of parents to avoid contamination between intervention parents who attend the same center (Murray, 1998). In addition, this approach allows centers and parents to be blind to condition until after parents complete the baseline assessment battery. For all participants, the assessment battery included parent-report questionnaires, an observation assessment and parent and toddler systemic cortisol both at baseline and 15 months later. Intervention participants commenced TOTS within 1 month of the baseline assessment. Questionnaires were also administered immediately post intervention for intervention participants.

The study was designed to adhere with the CONSORT statement (Schulz et al., 2010) and the intervention is reported using guidelines outlined with TIDieR (Hoffman et al., 2014).

### Ethics Approval and Clinical Trial Registration

Approval was obtained from two ethical standard bodies: The University of Melbourne Human Ethics Committee (#1443496), and the Department of Education and Training (#2015-002692). To ensure transparency, minimize bias, and improve reporting, the study was registered in the Australian and New Zealand Clinical Trials Registry (ACTRN12615000962538).

### Study Setting

The TOTS parenting program research trial was conducted in community settings within a 15 km radius west, north and east of Melbourne, Australia. Both daytime (10:00–12:00) and evening (19:30–21:30) parenting groups were offered to parents, however, the majority of parents indicated that an evening program was more suitable, and only two daytime groups were conducted. Venues included Mindful: Centre for Research and Training in Developmental Health (The University of Melbourne), council-funded community houses, and Child Care and Community Health Centers. Venues were chosen based on (a) proximity to the CC and MCH centers, ensuring that parents would not have to travel more than 8 km (b) free parking and (c) being accessible by public transport.

### Inclusion/Exclusion Criteria

Participants were eligible if they were a parent of an 18- to 36-month-old child attending CC or MCH centers. Exclusion criteria were insufficient English language skills to complete questionnaires and understand the program content. Language proficiency was established during the initial telephone conversation with the parent. Only one parent per child could attend so that at least one parent in each family learned the skills, however, it could be either parent participating.

#### Recruitment Procedure

Recruitment for this study occurred in several stages.

**Figure 1:**
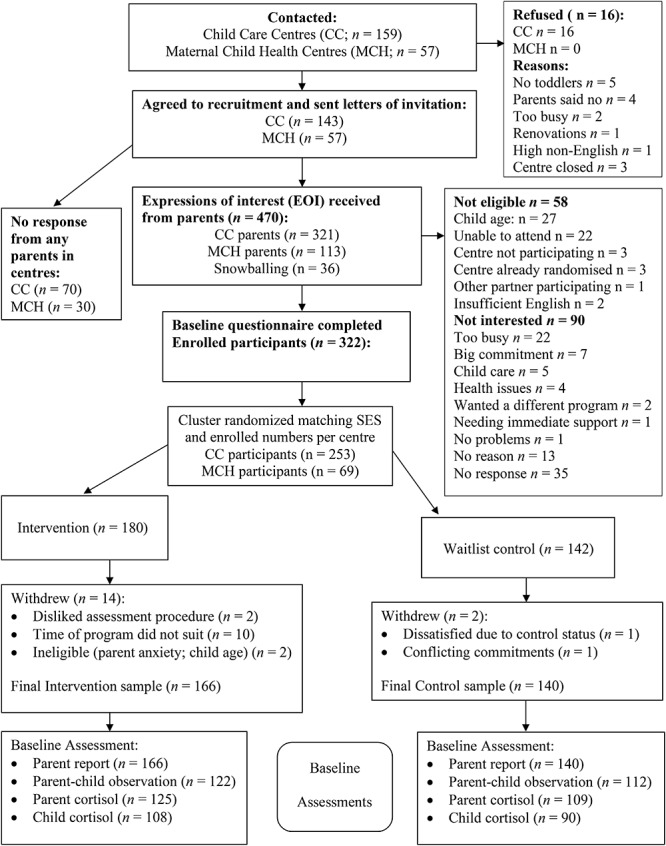
Recruitment process.

1. First, CC (*n* = 159) and MCH centers (*n* = 57) located in proximity to the program venue were approached and invited to participate.
2. Of centers approached, 16 CC (10%) declined and all MCH centers agreed (see Figure 1).

3. On two occasions, the project manager attended meetings of CC centers to discuss the study.
4. When centers consented, a TOTS research team member visited the center and provided an information pack, (i.e., a letter of invitation to the center director/MCH nurse, a plain language statement about the research, ethics approval letters, letter of invitation to parents, advertisement flyer, center consent forms, and parent questionnaire). Ten centers did not receive a visit and were happy to promote the program without a visit.
5. In consenting centers, parents of toddlers were invited to participate either via newsletter, billboard advertisement, or letters of invitation that were emailed or handed out. Parents directly contacted the research team via email or telephone.
6. Parents expressing interest participated in a telephone ‘intake interview’ conducted by a research assistant or student (with postgraduate psychology qualifications) to explain study processes and procedures.
7. Interested parents were emailed written information about the study, a consent form and a link to the baseline questionnaire (online, using Qualtrics). Questionnaires took approximately 45 min to complete and once submitted parents were officially enrolled in the study.
8. Parents deemed ineligible at this stage (e.g., child too old, insufficient English) were provided with referral options, or placed on a waitlist to participate in a control group program.
9. Following baseline questionnaire completion, parents attended an observation assessment with their toddler and to collect the hair sample/cortisol. Not all dyads attended an observation assessments due to limitations in funding. Selection for observation was random.

#### Randomization Procedure

Cluster randomization was conducted by pairing centers based on the median house price in that location and the number of parents of the center completing baseline questionnaires. Then each “center pair” was randomly allocated to either intervention or control using a computer randomization program, Research Randomizer (Urbaniak and Plous, 2015). Parents were informed of their condition (intervention or control) following completion of the baseline assessment.

### Study Retention

Retaining participants in a longitudinal intervention study, especially for those allocated to the 15-month waitlist control condition, can be a challenge (Heinrichs et al., 2005). Parents allocated to delayed start may find it difficult to wait, may withdraw or at times require immediate assistance. In the current study if concerns were expressed by parents about their child, TOTS research staff explored their concerns and discussed the strategies they were currently using to address the problem (i.e., no new strategies were suggested). Project staff engaged with the parent in a supportive way and offered reflective listening. If the parent was experiencing high distress, there were significant concerns about the parent or child’s wellbeing or there were concerns about the child or parent’s safety, then a referral for assistance was made.

Participants who withdrew from the study were contacted to establish the reason which was recorded along with the time of the withdrawal (e.g., after week 2 of the program, at follow-up, etc.).

### TOTS Intervention

*Tuning in to Toddlers* was adapted from the original TIK structured manual (Havighurst and Harley, 2010). The TOTS program is based on emotion socialization theory and draws on attachment, mindfulness and neurobiological theory and concepts. The program aimed to teach parents a modified version of the five steps of emotion coaching outlined by Gottman and DeClaire (1997): (a) be aware of low-intensity emotions in your child, (b) view your child’s emotions as a time for intimacy and teaching, (c) communicate understanding and acceptance toward your child’s emotions, (d) name the feeling, and (e) if necessary, give comfort, assist with choices, set limits, provide distraction, or problem solve. The program also includes activities designed to increase parents’ awareness, understanding and regulation of their own and their child’s emotions. This included a focus on family of origin experiences and exploration of attitudes toward emotions, teaching perspective taking and empathic reflective listening skills, and an aim to foster greater acceptance of emotions. Parents were also provided with information on cognitive, emotional and brain development in toddlers, and taught how to recognize and respond to their toddlers’ attachment and exploration needs. See Table 1 for description of program content. Delivery of program content was via psycho-education; watching DVD materials showing the difference between emotion dismissing and emotion coaching; exercises that scaffolded emotion coaching skill development; group discussions about application of emotion coaching with their own children as well as exploring parents beliefs about emotions and experiences in their family of origin with emotions such as sadness, fear, anger, jealousy, etc.; role-plays in demonstrations, with the whole group, using scripts and eventually without scripts in pairs; and homework activities where different content taught in sessions was put into practice at home. Refreshments were provided for group members to increase enjoyment in the group and to provide opportunities for building group cohesion.

**Table 1:** TOTS program content.

Session	Content
1) Setting out – how to raise emotionally intelligent children	Overview of program, Engaging participants and creating safe group space, Normalizing toddler behavior, Psychoeducation: emotional intelligence and emotion coaching, Role play contrasting emotion dismissing and emotion coaching using scripts, Home activities: notice low intensity emotions, naming emotions
2) Tuning in to your toddler’s emotions	Parent emotion awareness, Reflection on week and use of role plays, Meta-emotion: understanding influence of family of origin, Toddler development section, First 4-steps skills, Home activities: identify meta-emotion, first four steps, low intensity emotions
3) Understanding your toddler’s emotional experience	Guided relaxation, Reflection on week and use of role plays, Meta-emotion: identification of automatic reactions, Parenting styles, Developing empathy, Scaffolding being an emotion coach, Home activities: first four steps, building empathy
4) Self-care and exploring your toddler’s fears and worries	Guided relaxation, Parent self-care, Reflection on week and use of role plays, Meta-emotion: exploring family of origin experiences with fear, Fifth step of emotion coaching, Identifying attachment needs of toddler: connection and exploration, Coaching toddler’s to manage fears and worries, Home activities: connection/exploration to toddler, coaching fears/worries
5) Emotion coaching your toddler’s anger	Progressive muscle relaxation, Reflection on week and use of role plays, Meta-emotion: understanding your own anger and family of origin experiences with anger, Understanding causes of toddler’s anger, Responding to toddler’s anger, Home activities: building in a pause, emotion coaching anger
6) Emotionally intelligent parenting: now and in the future	Warm-up: relaxation or emotion awareness, Reflection on week and use of role plays, Emotional self-care and managing parent’s own anger, Review of five steps of emotion coaching, Role play with anger Sibling rivalry, Closing issues

*Tuning in to Toddlers* was delivered for 2 h/week across six, weekly sessions with two facilitators who used a structured facilitator manual. Parents were also provided with a TOTS participant workbook of all information and exercises delivered during the sessions. They were also provided with posters with a range of emotion faces and the emotion labeled underneath to assist the parent and child with emotional literacy. Nine intervention groups were facilitated by one of the TOTS team (Havighurst, Harley, Kehoe, and Wilson –all of whom had a M.A. or Ph.D. in Psychology or Education) who all had extensive experience in running the original TIK program and a volunteer co-facilitator who had been trained in TOTS. In addition, seven groups were co-facilitated by two registered psychologists originally trained in the TIK program, one who had completed a Ph.D. with the research team and one currently completing a Ph.D. Facilitators participated in supervision with the program authors throughout delivery.

### Intervention Groups

Intervention groups were scheduled to run in the evenings for 2 h per week during the last 6 weeks of each school term, except for two groups which were run in the morning. Groups had a minimum of 6 and a maximum of 14 parents. Attendance at the first session was flexible to allow for new group members to join in session two, however, after session two the group was closed, and no further participants were able to join. Parents were sent a confirmation letter with group location, dates and starting times and a reminder text message the day before the intervention started.

### Intervention Participant Retention

To retain families in the intervention, facilitators telephoned participants who missed sessions to explore reasons for absence and address potential barriers to attendance. While the intervention was provided free of charge, other barriers to attendance included a lack of transport, the need for childcare and anxieties about group participation. Attempts to address these barriers were made on a case-by-case basis to maximize participation.

### Intervention Implementation

Implementation was maximized using structured manuals and supervision and was measured on three dimensions: content fidelity, process fidelity, and attendance/dosage.

#### Content Fidelity/Intervention Adherence

Program facilitators followed a structured manual detailing how to deliver each session and each exercise. All facilitators were experienced in Tuning in to Kids (TIK) program delivery and were given additional training in the new TOTS intervention. They received fortnightly peer supervision to ensure program adherence. Facilitators completed a fidelity checklist after each group session to ensure core content of the intervention was covered, a method used in delivery of other interventions (Webster-Stratton et al., 2004). This checklist included: whether each exercise was completed; whether the psychoeducation component(s) was delivered; whether at least one demonstration of emotion coaching was presented to the group; whether a role play exercise in small groups was conducted; and whether homework assignments were given.

#### Process Fidelity

A questionnaire constructed to evaluate parents’ perceptions of the program was administered after completion of the intervention. Eleven items explored participants experience with the program, including: their perceptions of whether the program had been effective (notably whether they perceived their parenting to have changed or whether their toddlers were better able to express emotions); their satisfaction with the program; their confidence with responding to their child’s emotions; their ease/difficulty in learning the different skills (building in a pause to regulate their own emotions, capacity to ‘sit with’ and allow children to express emotions rather than going straight to distraction/problem solving); and their experience of the group process. Items were rated on a five-point Likert scale with space for adding comments.

#### Dosage

Facilitators recorded participant attendance to provide a measure of dosage.

### Measures

Parents were asked to complete a questionnaire battery, which included measures of demographic information, parent emotion socialization (emotion dismissing/coaching), parent functioning (their emotion regulation and mental health), toddler temperament and toddler social/emotional/behavioral functioning. In addition, observation assessments of parent–child interactions were conducted to measure parent emotion socialization. At the observation assessments hair samples were collected from parents and toddlers to measure stress cortisol.

### Parent-Report Questionnaires

#### Parenting

Two measures of parent-reported emotion socialization (primary outcome) were used, a measure of parental warmth, and a measure of hostile parenting (secondary outcomes).

*Parent Emotional Style Questionnaire* (PESQ) is based on the *Maternal Emotional Style Questionnaire* (MESQ; Lagacé-Séguin and Coplan, 2005), a parent-report questionnaire designed to measure emotion socialization beliefs. The original MESQ comprises 14 items measuring maternal beliefs about their child’s sadness and anger using a five-point Likert scale (ratings range from 1, *strongly disagree* to 5, *strongly agree*) for responses which are summed for Emotion Coaching and Emotion Dismissing sub-scales. Havighurst et al. (2010) added seven additional items to the MESQ to also include parent beliefs about children’s fears and worries and made the items parent gender neutral; this revised measure is referred to as the 21-item PESQ, and had good scale reliability in a community sample. The Emotion Coaching subscale included 11 items (e.g., *anger is an emotion worth exploring*). The emotion dismissing subscale included 10 items (e.g., *childhood is a happy-go-lucky time, not a time for feeling sad or angry*). Havighurst et al. (2010) also selected the five PESQ items that tapped parents’ empathy and emotional connection with their child (e.g., *when my child is scared, it’s an opportunity for getting close; when my child is angry, I take some time to try to experience this feeling with him/her*) to create a subscale of Empathy. For the present study, Cronbach’s alphas at baseline were α = 0.79 for Emotion Coaching, α = 84 for Emotion Dismissing, and α = 0.71 for Empathy.

*Coping with Toddlers Negative Emotions Scale* (CTNES) – Parents’ responses to toddlers’ negative emotions were assessed using the CTNES (Spinrad et al., 2004), an adaptation of the Coping with Children’s Negative Emotions Scale (CCNES; Eisenberg et al., 1996). Parents read 12 hypothetical scenarios where their toddler is distressed, angry or upset (e.g., *If my child is going to spend the afternoon with a new babysitter and becomes nervous and upset because I am leaving him, I would*…), and are asked to rate the likelihood of responding to the situation in each of seven possible ways. Ratings range from 1 (*very unlikely*) to 7 (*very likely*). Scores are calculated for the seven subscales of: (a) Distress Reactions (e.g., *feel upset or uncomfortable because of my child’s reactions*); (b) Granting the Child’s Wish (e.g., *change my plans and decide not to leave my child with the sitter*); (c) Problem-Focused Reactions (e.g., *help my child think of things to do that will make it less stressful, like me calling him once during the evening*); (d) Emotion-Focused Reactions (e.g., *distract my child by playing and talking about all of the fun he will have with the sitter*); (e) Expressive Encouragement (e.g., *tell my child that it’s ok to be upset*); (f) Punitive Reactions (e.g., *tell my child he won’t get to do something enjoyable…if he doesn’t stop behaving like that*); and (g) Minimizing Reactions (e.g., *tell my child that it’s nothing to be upset about*). The CTNES has demonstrated predictive validity and adequate test–retest reliability from 2 to 4 months (Spinrad et al., 2007). In the current study, the respective Cronbach’s alphas at baseline were α = 0.79 for Distress Reactions, Granting the Child’s Wish α = 0.79, Emotion Focused Reactions α = 0.76, Expressive Encouragement α = 0.94, Problem focused Reactions α = 0.85, Punitive Reactions α = 0.82, and Minimizing Reactions α = 0.86.

##### Parental warmth

Nine items were used to measure parent-reported warmth from the *Longitudinal Study of Australian Children* study (Growing up in Australia, 2017) with a five-point Likert scale, (e.g., *Hug or hold my toddler for no particular reason*, or *Feel close to my toddler both when he/she was happy and when he/she was upset*). Items were summed to give a total score (range 0–40). Cronbach’s alpha for this scale at baseline was 0.81.

##### Hostile parenting

Five items from a Hostile Parenting scale used in the *Longitudinal Study of Australian Children* (Growing up in Australia, 2017) were used to assess angry responses to difficult child behaviors. Parents are asked to rate on a five-point Likert scale the frequency of specific behaviors in the previous 4 weeks (e.g., *I have been angry with my toddler*, or ‘*I have lost my temper with my child*). Items were summed (range 0–20) with higher scores indicating more hostile parenting. Cronbach’s alpha for this scale at baseline was α = 0.88.

### Parent Functioning

A measure of parent emotion regulation and a mental health screen were used to capture parent functioning as a secondary outcome of the intervention.

*Difficulties with Emotion Regulation Scale* (DERS) – The DERS is a 36-item parent-report questionnaire measuring parent’s emotional awareness and regulation difficulties (Gratz and Roemer, 2004). Items are rated from 1 (*almost never*) to 5 (*almost always*), with higher scores indicating greater difficulties. The DERS is comprised of six subscales measuring: (a) emotional awareness (e.g., *I pay attention to how I feel*); (b) emotional non-acceptance (e.g., *When I’m upset, I become embarrassed for feeling that way*); (c) emotional clarity (e.g., *I have difficulty making sense out of my feelings*); (d) capacity to undertake goal-directed behavior when distressed (e.g., *When I’m upset, I have difficulty getting work done*); (e) impulse control (e.g., *When I’m upset, I feel out of control*); and (f) access to regulation strategies (e.g., *When I’m upset it takes me a long time to feel better*). The total scale score was computed for use in the current study as an indicator of overall difficulties in emotion awareness and regulation. The DERS total score has demonstrated high internal consistency (Cronbach’s alpha = 0.93), convergent and predictive validity, and good test–retest reliability over 4–8 weeks (Gratz and Roemer, 2004). In the current study, Cronbach’s alpha for the total score at baseline was 0.94.

*The Kessler 6* (Kessler et al., 2002) was used to measure parent mental health, a 6-item parent-report questionnaire measuring how often parents felt, e.g., *hopeless*, *restless*, and *worthless* in the past 30 days. Items are rated on a five-point Likert scale from *none of the time* to *all of the time* and summed to provide a total score, with higher scores indicating greater psychological distress. The Kessler-6 has demonstrated excellent internal consistency, with a reported Cronbach’s alpha of 0.89 (Kessler et al., 2002). In the current study, Cronbach’s alpha at baseline was 0.80.

### Toddler Functioning

Toddler behavior was measured as a secondary outcome, while toddler temperament and developmental status were measured as possible confounding factors.

*Brief Infant-Toddler Social Emotional Assessment* (BITSEA; Briggs-Gowan et al., 2002) – Toddler social-emotional/behavioral problems were measured using the BITSEA, a 34-item parent-report measure which assesses toddler behavior problems occurring in the past 4 weeks. Items (e.g., *Is restless and can’t sit still*) are rated from 1 (*not true/rarely*) to 3 (*very true/often*) and measures internalizing problems (e.g., *seems nervous, tense or fearful*, or *worries a lot*), externalizing problems (e.g., *hits/shoves, kicks or bites children*, or *cries or tantrums until exhausted*), and dysregulation problems (e.g., *has trouble falling asleep*, or *gags or chokes on food*). The measure has demonstrated excellent test–retest reliability, good interrater agreement across parents and carers with this age group (Briggs-Gowan et al., 2004; Zimmer-Gembeck and Thomas, 2010), and has been used in an Australian context (e.g., Zimmer-Gembeck and Thomas, 2010). Briggs-Gowan et al. (2004) report Cronbach’s alpha for this subscale as 0.79 for parent ratings. In the current sample, Cronbach’s alpha at baseline for the total score was 0.74.

*Toddler Temperament Scale* (TTS) – Toddler temperament was measured using the *Short Infant Toddler Temperament Questionnaire*, developed by the Australian Temperament Project (ATP; Prior et al., 1989). This parent-reported scale consists of 13 items and captures three dimensions of temperament (Approach, Reactivity, and Persistence), giving a subscale for each, as well as a total score. Parents report the frequency with which their toddler, e.g., *smiles when an unfamiliar adult plays with him/her* (Approach), *responds to frustration intensely (screams, yells)*, (Reactivity), and *stops to examine objects thoroughly (5 min or more)* (Persistence), on a six-point Likert scale from *almost never* to *almost always*. A TTS total score was computed to reflect a more difficult temperament (higher score) using Reactivity and reverse scoring Approach and Persistence. The TTS has demonstrated satisfactory reliability and validity and has been used widely in Australia (e.g., Pedlow et al., 1993). In the current study, Cronbach’s alpha for the total difficulty scale at baseline was 0.67.

*Parents’ Evaluation of Developmental Status* (PEDS) is a parent-reported 19-item screening measure that used a five-point Likert scale (from *never* to *almost always)* for detecting developmental and behavioral problems in children aged from birth to 7 years and 11 months. The measure was developed and validated in the United States but has also been validated in Australia where studies show it is has reliability (Glascoe, 1998). In the current study, Cronbach’s alpha at baseline was 0.82. Six additional items were used from the PEDS to assess Expressive and Receptive speech and language in the toddler. Items were rated on a three point Likert scale (*never, sometimes, always*) and included, *Carry out a simple instruction, Ask for a request to be repeated, Follow a conversation, Pass on a simple message, Clearly explains things*, and *Uses speech that is easily understood*. In the current study, a total score for Expressive/Receptive Language was computed and Cronbach’s alpha at baseline was 0.79.

### Observation Assessment

A video-recorded observation assessment (conducted by a research assistant blind to the intervention condition of the dyad) was used to measure parent emotion socialization as a primary outcome to enable observation of the dyad during a series of unstructured and structured activities. These were designed to observe the parent and child playing together, to create opportunities for parents to talk with their toddlers about emotions, to elicit emotions in the toddler so that the parent’s responses could be observed and to observe the dyad in terms of mutually responsive orientation. Structured coding will be used to quantify these constructs.

#### Structured Observation Tasks

Parents and toddlers entered a room with a camera set up and a one-way mirror. The assessment began with a free play task without toys (5 min), followed by free play with a doll’s house/furniture, a toy car and toy family members (5 min). Parents were then asked to complete a semi-structured story telling task using a doll’s house where parents were given instructions to use the play equipment to tell a story with four different events where the: parents go away overnight leaving children in care of a trusted adult; children have a fight; the family dog runs away; and the parents return the next day (Cervantes and Callanan, 1998). Next parent and toddler selected two emotion faces (from a selection of five faces of toddlers expressing different emotions) and were asked to discuss a time when the child had felt this way. Then, a frustration task was given where a desirable toy in a plastic see-through bag was placed in front of the child and the parent was instructed to fill in a questionnaire before the child could open the desirable toy. The child was given three simple wooden blocks to play with while waiting. Once the parent completed the questionnaire, the toddler could play with the toy for 5 min before they were asked to pack up.

#### Coding

Three methods of coding will be used that capture different aspects of emotion socialization and emotional responsiveness. Observed emotion-coaching and emotion-dismissing behaviors will be coded using a structured manual of global ratings developed by Baker et al. (2011). Emotion coaching comprises of five subscales: (a) structuring, (b) sensitivity, (c) validation/encouragement of emotions, (d) enthusiasm, and (e) intimacy/warmth/affection. Emotion dismissing comprises four subscales: (a) derogation, (b) intrusiveness, (c) minimization/discouragement of emotions, and (d) detachment/disinterest. These dimensions will all be rated on a five-point Likert scale and averaged to give overall scores. This coding manual was used in the pilot study of TOTS (Lauw et al., 2014) where inter-rater differences of only one point difference were present for only 15 of 64 variables, indicating high inter-rater reliability.

Additional aspects of emotion coaching will be coded that include whether the parent engages in key components of emotion coaching outlined by Gottman et al. (1996) that include noticing, connecting, reflecting, empathizing, problem solving/limit setting, timeliness of responses, and how parents regulate their emotions during the observation (Havighurst et al., 2017).

Kochanska’s Mutually Responsive Orientation (MRO; Kochanska, 2017, Personal Communication) will be coded where dyads are rated on a continuum from low to high on a five-point Likert scale ranging from 1 = *very untrue* to 5 = *very true.* Those low on MRO would be coded as *adversarial, disconnected, unresponsive, hostile, and affectively negative*. Those high on MRO would be coded as *mutually responsive, coordinated, harmonious, in sync, attuned to each other, mutually cooperative, and affectively positive*.

#### Coders and Training

Coding will be carried out by undergraduate and graduate psychology students external to the study and blind to participant intervention status. All students will participate in 2 days of training. Interrater coding will be completed with the first and second author until student coding is moderate to high in terms of interrater reliability. During coding students will have regular supervision to maximize interrater reliability and address difficulties with coding. Interrater reliability will be conducted with 10% of all observation assessments.

### Stress Cortisol

Cortisol in hair samples will be collected to measure parent and toddler stress as a secondary outcome. To use a physiological measure to assess parental and toddler stress levels, hair cortisol assays using hair cut from the head will be collected from parents and toddlers at the time of the observation assessment. This technique has been validated in human studies and has also been found to be a marker of chronic systemic stress (Russell et al., 2012). With hair growing at approximately one centimeter/month, the first centimeter of hair from the scalp can be measured to determine the previous months’ cortisol production.

Following a written protocol, researchers cut a sample of hair approximately 3 mm in width from the back of the parent and toddler’s head as close to the scalp as possible. Samples will then be tied using cotton and packaged in foil for storage at room temperature. Analysis will be conducted by an external laboratory, Stratech Scientific. Analysis involves removal of surface contaminants by washing samples with isopropanol for 3 min on a rotor then left to dry in a clean protected hood for 4 days. Hair will then be stretched out and 1 cm of hair (25–40 mg, representing cortisol levels during the last month) will be cut from the scalp end and ground to a fine powder. Steroids will be extracted with methanol. The extracted slurry will be centrifuged at high speed to pellet hair particles and clear methanol containing extracted steroids will then be transferred to a clean tube and evaporated. Assay diluent buffer will be placed in each tube, vortexed and left to stand then vortexed again. To optimize accuracy each sample analysis will be duplicated. Cortisol will be quantified using ELISA kits (Salimetrics, United States). Intra assay variability will be reported at 5.1% and inter assay variability at 5.8%. Data will be provided as nanograms of cortisol per 50 mg of hair.

### Power Analyses

Using methods proposed by Eldridge et al. (2006), the estimated design effect for the study is 1.30 (based on 40 clusters of average size 7, a coefficient of variation for cluster size of 0.25, and a conservative at worst intra-class correlation of 0.05). The following power calculation incorporates this estimated design effect from clustering, and is done for feasible differential treatment effects at 15 months follow-up, where attrition will be highest. The treatment effect difference is conservatively estimated to result in group differences of about 0.40 SDs for continuous outcome measures. To achieve 80% power at a 5% significance, the required sample size at follow-up needs to be 100 × 1.30 = 130 participants in each condition. Based on our other studies, including our pilot study, we estimated there will be 10% attrition. This implies that 130/0.90 = 144 participants are needed in each condition for the study to have sufficient power.

### Planned Statistical Analyses

Data will be analyzed using SPSS, beginning with data cleaning and assumption testing. Multiple imputation will be used to address missing data. A series of analyses are planned. First, independent samples *t*-test analyses will determine whether there are any systematic differences between pre-intervention scores for demographic and outcome variables for the intervention and the control conditions. Any baseline differences will be used as covariates in subsequent analyses. In addition, those with missing time 2 and 3 data points will be examined to determine differences from those completing follow-up assessment. Next, because parents are nested in centers, intraclass correlations will be computed to assess if center membership contributes to outcome variance. Our previous work has shown that kindergarten or school membership contributes from 8 to 11% of variance in child outcome variables (e.g., Havighurst et al., 2010; Wilson et al., 2012). Therefore, multilevel analyses using the mixed procedure in SPSS will be conducted to compare intervention and control participants across the different measures over time whilst considering the impact of the nested design (i.e., children attending different child care centers) and baseline covariates. Effect sizes will be calculated using Cohen’s *d*.

## Results

A total of 470 parents of toddlers expressed interest in participating in the study (see Figure 1, participant flow). An exact percentage uptake rate was difficult to determine for MCH centers because advertising was on the walls of centers and it was not possible to determine the number of times the nurses offered the program to parents. However, CC centers had an average of 65 toddlers per center – resulting in a 4.6% uptake. Parents had heard about the study via CC (*n* = 321), MCH (*n* = 113) centers, or other participating parents (*n* = 36; note that these were enrolled only if their center was participating in the study). Of the 470 parents, 58 participants were not eligible to take part, and 90 parents withdrew their interest from the study, leaving a total of 322 parents who completed their baseline questionnaire and were enrolled in the study (68.5%). Of the 58 participants who were not eligible to take part, 27 parents had children not within the specified age range (too old *n* = 19; too young *n* = 8), 22 parents were unable to attend the designated group (due to location or times not suitable *n* = 17, moving away *n* = 4, or taking a long vacation *n* = 1), two parents had insufficient English, and one parent was the ex-partner of a parent in the study. Three parents’ centers were not part of the study, and three parents expressed interest after their center was matched and randomized and so were not included in the final sample. All parents not eligible were offered referrals to other parenting programs. Of the 70 parents who did not go ahead with participating after expressing interest, the majority withdrew their interest because they were too busy (*n* = 22) or the time commitment was too great (*n* = 7). Other reasons for not going ahead with the study were child care issues (*n* = 5), health reasons (*n* = 4), needing immediate support (*n* = 1), deciding they no longer needed parenting help (*n* = 1), and wanting to do a different program (*n* = 2). Thirteen parents did not provide a reason for withdrawing after expressing interest, and 35 parents did not respond to calls or emails.

Three hundred and twenty-two participants completed baseline questionnaires with 180 in the intervention condition and 142 allocated to the waitlist control condition. Of the 322 enrolled participants, 16 parents withdrew after completion of their baseline data. Of these, two were waitlist control parents (one did not wish to wait 15 months; the other had conflicting commitments in 15 months). Of the 14 intervention parents, two were excluded because they were assessed to be ineligible (e.g., not suitable for the parenting program due to high social anxiety *n* = 1; child too young *n* = 1). Seven parents did not attend any sessions (baby too unsettled *n* = 1, child care arrangements did not work out *n* = 3, unforeseen traumatic event *n* = 1, other commitments *n* = 2). Two intervention parents found the observation assessments stressful and did not wish to participate in the intervention. A scheduled daytime group did not go ahead due to low numbers and three of these participants were unable to attend an evening session. Instead they were sent a DVD and program booklet and were offered the program at a future date.

The final sample was 306 participants (166 intervention and 140 waitlist control), of which 241 were recruited from CC and 65 from MCH centers. Observation assessments were conducted with a random selection of 246 parent–child dyads at baseline (as mentioned earlier this was due to funding constraints). Hair cortisol concentration data was collected for 235 parents who attended the assessments and for 201 toddlers at baseline. Hair samples were not taken from 11 parents due to them choosing not to give a hair sample and from 34 toddlers due to parents not wishing the hair sample to be taken or due to toddler’s being distressed at receiving a haircut (some had never had a haircut before). There were no significant differences between those participants who completed the questionnaires only and those who completed the full assessment battery (including questionnaires, parent–child assessment and cortisol stress measures).

Participants were 272 mothers (88.9%), one kinship caring grandmother (0.3%), and 33 fathers (10.8%) with a mean age of 37.1 years (*SD* = 4.9; range = 16.9–70.1 years) of a toddler aged on average 25.6 months (*SD* = 5.3; age range = 17.2–36.4 months; males *n* = 176; 54.6%). The majority of participants had either one (*n* = 176; 57%) or two children (*n* = 115; 37%; range = 1–5 children), and resided with their child and the child’s other parent (*n* = 270; 88.2%), while six parents had re-partnered (2%), 24 (7.8%) were sole parents, and six lived in shared custody arrangements (2%). English was the main language spoken at home (93.1%), with 224 participants (73.2%) born in Australia, and the remainder born in North America/New Zealand/United Kingdom/Europe (39 participants; 12.7%) or Asia/Africa/Middle East (29 participants; 9.5%). High school completion rate was 95.4%, and most parents reported completing a post-school qualification (96.4%; none = 3.6%; certificate/trade = 5.6%; diploma = 5.9%; undergraduate degree = 39.5%; higher degree/diploma = 44.2%). Two hundred and thirty-two parents (76.1%) were employed (77.8%), working an average of 26.8 h per week (*SD* = 10.02; range 2–50 h per week), and five parents (1.6%) were on maternity leave. Occupations were mainly managerial or professional (56.9%), but also included associate professionals (9.5%), clerical/sales/transport workers (8.1%) and tradespersons (1.3%). Seventy-three participants (23.9%) did not provide details about their occupation. Few families (5.6%) were below the Australian poverty threshold of AUD $49,972 per year for couples with two children (Melbourne Institute of Applied Economic and Social Research, 2015). More specifically, 37 parents (12.1%) reported low gross annual family income ($0–64,999), 86 parents (28.1%) reported middle-incomes ($65,000–119,999), and 183 (59.8%) reported higher incomes (>$120,000). In 2016, the median household income for Melbourne was $95,000 (Australian Bureau of Statistics, 2016).

## Discussion

The toddler years are a time when children flourish in their emotional and social development and often begin to assert their independence. Their limited language and underdeveloped emotion regulation skills often mean they experience intense emotions and dysregulated behavior creating frequent challenges for their parents. The way in which parents manage their own emotions during their experience of parenting, and how they respond to and teach their children about emotions plays a key role in shaping the child’s emotional competence (Eisenberg et al., 1998). TOTS is a parenting program that aims to help parents learn skills to assist in these aspects of parenting. The current paper outlined the methodology and recruitment strategy being used in a randomized controlled trial of TOTS where parents were offered a group parenting program to learn skills in emotion coaching and parent emotion awareness and regulation. At a time when young children are developing so rapidly, efforts to reduce dismissive or critical parenting and enhance emotion coaching parenting are likely to have important long-term effects on children’s emotional competence.

A Cochrane review (Barlow et al., 2016) of group parenting programs for parents of children up to 4 years of age has shown that the existing evidence is somewhat poor in quality for universal or at-risk families and it is not clear whether interventions with this age group are effective. However, this has predominantly been due to the small number of evidence-based programs and intervention studies for this target age group. Our own experience with the challenges with recruitment of parents with young children may, in part, provide some insight into the reasons there are so few evidence-based programs for parents of toddlers.

The first major challenge for engaging participants and reducing barriers to participation was in how to reach parents of young children. CC and MCH centers were the target for recruitment (as two universal services seeing parents of young children) and both are places where support with parenting may be sought in the early years. Some participants also came through snowballing (word of mouth) and Facebook/other social media after hearing about the study from other parents at their center. This last method of recruitment is important because parents seek out and obtain considerable information about parenting from peers and social media (Akers and Gordon, 2018).

The second major challenge for recruitment was how to motivate and engage parents of toddlers to attend a parenting program. Many parents were struggling with the challenges of parenting a young family. At the same time, weekly attendance at a parenting program was difficult unless the parent was well organized so that they could leave the house at bedtime, had a supportive partner or family to care for the children, and was able to think beyond the immediate ‘survival’ of parenting young children. Many parents had a new born as well as a toddler, and managing the competing needs of two or more young children can be very challenging. Offering day time groups did not improve parent engagement with only 2-day time groups being run due to the small number of parents selecting this time slot. Offering childcare might also have assisted with daytime group attendance, however, in our pilot study no parent expressed interest in this option with reports that the children were too young for childcare with relative strangers for such a short duration. As a result, the sample that were recruited for this study were predominantly from middle class families, perhaps due to the resources (practical and emotional) required to attend a parenting program. Future research with parents of toddlers to explore the barriers for participation in parenting programs would be very useful.

A search of the literature on parenting programs for young children is striking in the lack of evidence based programs with community samples (for example Barlow et al., 2016). This may be because parents do not seek external support and parent education unless in crisis (hence many evidence-based programs are targeted to participants with clinical-level difficulties) or because parents do not seek this input until their children are older and family life becomes more stable. We had an average uptake of 4.5% of parents expressing interest from CC and less from MCH centers. This was lower than our previous efficacy and effectiveness trials of the *Tuning in to Kids* parenting program where recruitment of community samples of parents of 4- to 5-year-old children resulted in 10% recruitment rates (Havighurst et al., 2010; Wilson et al., 2012). This higher rate of recruitment has also been reported in other research trials of programs for parents of school aged children and adolescents in community settings (Kehoe et al., 2014). It may be that recruitment of parents of toddlers is harder regardless of efforts to overcome barriers. Future efforts to understand and overcome barriers to participation might include offering a program with a shorter duration of delivery, a spread of sessions over time or using an online platform where parents can access the intervention without requiring childcare.

### Strengths and Limitations

This study is innovative in targeting parents when their children are young and addressing emotion socialization processes rather than behavioral methods of parenting. The study will follow the CONSORT guidelines for intervention evaluation (Schulz et al., 2010) and will use mixed methods of evaluation (parent-report, parent–child observation, physiological measures of systemic cortisol in parent/toddler) in order provide detailed analysis of the potential impact of the intervention on primary and secondary outcomes and to reduce expectancy bias that can occur by using parent report alone. Cluster randomization of centers not individuals enabled snowballing/social media to increase recruitment in any particular center while ensuring that condition assignment was unknown to participants and the research team during recruitment. Cluster randomization also enables the effect of centers to be taken into account in statistical analyses by computing intra-class correlations and including these in multilevel analyses of outcomes. Immediate and long-term follow-up of 12 months post intervention (15 months from baseline) will be used for examining changes over time and whether the intervention achieves the intended primary and secondary outcomes of improving parenting, parent functioning, and children’s wellbeing. Measures of implementation quality, including fidelity of intervention delivery and post-intervention satisfaction by parents will enable detailed information to be collected about the intervention and potential barriers to retention.

In terms of limitations, delivery of the intervention to control participants prohibits the possibility of long-term evaluation of outcomes. Melbourne is a highly culturally diverse city, however, provision of the intervention and measures only in English will reduce the generalizability of the outcomes to families from diverse cultural backgrounds. As discussed above, participation in the trial is contingent on parents being able to attend 6 × 2 h parenting sessions: the resources required to be able to do this as a parent of young children mean that the sample is skewed to those from higher SES and education background with more resources. The generalizability of the intervention and the study findings to lower SES families where such resources may not be available will need to be made with caution. Despite these limitations, the paucity of research with universally offered group programs for parents of toddlers mean this study makes an important contribution to the empirical field.

## Conclusion

The current study offers important information about a new emotion socialization program for parents of toddlers. The protocol outlines a method adhering to the CONSORT guidelines about conducting an efficacy trial under optimal conditions whilst also addressing the significant barriers to engagement of parents of young children in group-delivered parenting programs. Outcomes of the trial will provide important information about whether the program works and for whom.

## Trial Status

At the time of submission (December, 2018), 306 parents of toddlers had been recruited.

## Ethics Statement

This study was carried out in accordance with the recommendations of The University of Melbourne Human Ethics Committee (#1443496), and the Department of Education and Training (#2015-002692). All subjects gave written informed consent in accordance with the Declaration of Helsinki. The study protocol was registered in the Australian and New Zealand Clinical Trials Registry (ACTRN12615000962538).

## Author Contributions

All authors contributed to design of this study and writing of this manuscript. SH, CK, and AH were involved in the intervention development and delivery. SH, CK, and AJ were involved in conducting the research.

## Conflict of Interest Statement

SH, CK, and AH wish to declare a conflict of interest in that they may benefit from positive reports of this program. The remaining authors declare that the research was conducted in the absence of any commercial or financial relationships that could be construed as a potential conflict of interest.
